# A Copula Based Approach for Design of Multivariate Random Forests for Drug Sensitivity Prediction

**DOI:** 10.1371/journal.pone.0144490

**Published:** 2015-12-10

**Authors:** Saad Haider, Raziur Rahman, Souparno Ghosh, Ranadip Pal

**Affiliations:** 1 Department of Electrical and Computer Engineering, Texas Tech University, Lubbock, Texas, United States of America; 2 Department of Mathematics and Statistics, Texas Tech University, Lubbock, Texas, United States of America; Koc University, TURKEY

## Abstract

Modeling sensitivity to drugs based on genetic characterizations is a significant challenge in the area of systems medicine. Ensemble based approaches such as Random Forests have been shown to perform well in both individual sensitivity prediction studies and team science based prediction challenges. However, Random Forests generate a deterministic predictive model for each drug based on the genetic characterization of the cell lines and ignores the relationship between different drug sensitivities during model generation. This application motivates the need for generation of multivariate ensemble learning techniques that can increase prediction accuracy and improve variable importance ranking by incorporating the relationships between different output responses. In this article, we propose a novel cost criterion that captures the dissimilarity in the output response structure between the training data and node samples as the difference in the two empirical copulas. We illustrate that copulas are suitable for capturing the multivariate structure of output responses independent of the marginal distributions and the copula based multivariate random forest framework can provide higher accuracy prediction and improved variable selection. The proposed framework has been validated on genomics of drug sensitivity for cancer and cancer cell line encyclopedia database.

## Introduction

An important goal of systems medicine is to generate genomics informed personalized therapeutic regimes with higher efficacy. The ability of inferred models to accurately predict sensitivity of an individual tumor to a drug or drug combination can assist in designing personalized cancer therapy treatments with expected effectiveness significantly higher than current standard of care approaches. A variety of techniques have been proposed for drug sensitivity prediction based on genetic characterizations. A common approach is to consider a training set of cell lines with experimentally measured genomic characterizations (RNA expression, Protein Expression, Methylation, SNPs etc.) and response to different drugs, and design supervised predictive models for each individual drug based on one or more genomic characterizations. For instance, statistical tests have been used to show that genetic mutations can be predictive of the drug sensitivity in non-small cell lung cancers [1]. In [2], gene expression profiles have been used to predict the binarized efficacy of a drug over a cell line with an accuracy ranging from 64% to 92%. In [3], a co-expression extrapolation (COXEN) approach was used to predict the drug sensitivity for samples outside the training set with an accuracy of around 82% and 75% in predicting the binarized sensitivity of bladder and breast cancer cell lines respectively. Tumor sensitivity prediction has also been considered as (a) a drug-induced topology alteration [4] using phosphor-proteomic signals and prior biological knowledge of generic pathway and (b) a molecular tumor profile based prediction [1, 5]. Drug sensitivity prediction using an elastic net regression analysis [6] over more than 100,000 genomic features (RNA expression, Mutational status of specific genes and SNPs) was considered in [7]. The correlation coefficients between the predicted and actual sensitivity over 450 cell lines using 10 fold cross validation ranged from 0.08 to 0.76 for different targeted drugs. [8] used a Random Forest (RF) based approach to tumor prediction in the NCI 60 cell lines with performance exceeding multiple existing approaches.

The motivation towards Multivariate random forests originated from our participation in a recent community based effort organized by Dialogue on Reverse Engineering Assessment and Methods (DREAM) project [9] and National Cancer Institute (NCI) that explored multiple different drug sensitivity prediction algorithms applied to a common dataset. More than 40 different approaches were applied and our submission based on Random Forests (RF) that considered the generation of individual models for each drug was a top performer in the challenge [10]. However, sets of drugs can have common targets or paths of action resulting in correlated responses in their sensitivities, which can possibly be utilized to improve the accuracy of the supervised predictive model. Note that the best performing approach in this challenge considered the relationships in the output responses in the form of Bayesian multitask learning [9] and the details of this multi-output regression approach is available at [11]. Multi-drug model has also been pursued by [12] where they have used multi-output regression using neural networks on the Genomics of Drug Sensitivity for Cancer (GDSC) dataset. Since our top performing RF model was ignoring the multi-drug response dependencies, we investigated the extension of the RF framework to Multivariate Random Forests (MRF) that incorporates the relationships between the output sensitivities. The objective of the MRF framework is to generate predictions that minimize error and have a multivariate structure similar to the relationships in the original training output responses. To generate individual multivariate regression trees for the construction of MRF, we altered the node cost function to consist of the weighted sum of the squared differences from the mean (similar to univariate regression tree cost function) and a penalty term to capture the difference between the multivariate relationship in the output responses at the node and the multivariate relationship observed in the original training data. Our initial choice for creating the regression tree node cost was to use Mahalanobis distance square [13], which improved our results as compared to RF approach [14]. The Mahalanobis distance square, being based on the covariance of the output responses, is suitable for scenarios where the relationships between the drug sensitivities is linear but can fail to capture non-linear relationships with low correlation coefficients. With this consideration, this article explores the design of multivariate regression trees that can capture all types of relationship in the output responses.

To capture the multivariate structure present in the output responses, we consider the use of copulas as they can deconstruct a multivariate distribution into its marginal distributions and underlying relationships that are represented by copula functions. We expect that the multivariate distribution of the sensitivities to a drug set will change based on the type of cell lines they are being applied to but the relationship structure separated from the marginal sensitivity distributions will remain similar. As an example, consider two drugs Gefitinib and Lapatinib that might have higher sensitivities when applied to breast cancer cell lines but lower sensitivities when applied to brain tumor cell lines. Thus, the multivariate distribution representing the sensitivities to the two drugs will appear to be skewed towards higher values for Breast cancer cell lines and skewed towards lower values for Brain tumor cell lines. However, we might observe similar correlation coefficients between the sensitivities for the Breast cancer cell lines and the Brain tumor cell lines as the primary target of both the drugs (EGFR) maintains the relationship. The correlation coefficient is one of the measures of the multivariate structure that will mostly capture linear relationships. However, incorporating the ability to separate the marginal distributions from the multivariate distribution will provide us with a more detailed representation of the underlying associations.

In this article, we discuss the appropriateness of copulas for capturing the multivariate structure in output responses and subsequently propose a cost function utilizing copulas for evaluating multivariate regression tree node splits. The cost function is a weighted combination of (a) the sum of squares of the differences between the node and mean responses and (b) the difference in the empirical copula observed at the node and the copula representing the training samples. We also demonstrate the suitability of the framework in variable selection where it provides higher importance to biologically relevant features as compared to competing approaches.

Note that the generation of the node cost function based on copulas presented in this paper can be considered as a generalization of the Multivariate Random Forest framework based on the square of Mahalanobis distances [13]. The presented approach can be applied to any predictive modeling scenario with multiple interrelated output responses.

The paper is organized as follows: The *Methods* section provides a description of the Random Forest framework with proposed extensions to copula based Multivariate Random Forests including design of the node cost function and an illustrative example. The *Results* section contains the performance of the proposed approach when applied to Genomics of Drug Sensitivity in Cancer database. The *Conclusions* section presents the conclusions of the current study and discusses future directions.

## Methods

We first present a description of Random Forest regression followed by extension to Multivariate Random Forest regression utilizing the covariance structure of the data. Subsequently, the concept of Copulas is introduced along with their application in designing node splits for multivariate regression trees.

### Random Forest Regression

Random Forest (RF) regression refers to ensembles of regression trees [15] where a set of *T* un-pruned regression trees are generated based on bootstrap sampling from the original training data. For each node, the optimal node splitting feature is selected from a set of *m* features that are picked randomly from the total *M* features. For *m* ≪ *M*, the selection of the node splitting feature from a random set of features decreases the correlation between different trees and thus, the average response of multiple regression trees is expected to have lower variance than individual regression trees. Larger *m* can improve the predictive capability of individual trees but can also increase the correlation between trees and void any gains from averaging multiple predictions. The bootstrap resampling of the data for training each tree also increases the variation between the trees.

### Process of splitting a node

Let *x*_*tr*_(*i*, *j*) and *y*(*i*) (*i* = 1, ⋯, *n*;*j* = 1, ⋯, *M*) denote the training predictor features and output response samples respectively. At any node *η*_*P*_, we aim to select a feature *j*_*s*_ from a random set of *m* features and a threshold *z* to partition the node into two child nodes *η*_*L*_ (left node with samples satisfying *x*_*tr*_(*I* ∈ *η*_*P*_, *j*_*s*_)≤*z*) and *η*_*R*_ (right node with samples satisfying *x*_*tr*_(*i* ∈ *η*_*P*_, *j*_*s*_)>*z*).

We consider the node cost as sum of square differences:

$$D\left(\eta_{P}\right)=\sum_{i\in \eta_{P}}{\left(y\left(i\right)-\mu \left(\eta_{P}\right)\right)}^{2}$$ (1)

where *μ*(*η*_*P*_) is the expected value of *y*(*i*) in node *η*_*P*_. Thus the reduction in cost for partition *γ* at node *η*_*P*_ is

$$C\left(\gamma ,\eta_{P}\right)=D\left(\eta_{P}\right)-D\left(\eta_{L}\right)-D\left(\eta_{R}\right)$$ (2)

The partition γ* that maximizes *C*(*γ*, *η*_*P*_) for all possible partitions is selected for node *η*_*P*_. Note that for a continuous feature with *n* samples, a total of *n* partitions needs to be checked. Thus, the computational complexity of each node split is *O*(*mn*). During the tree generation process, a node with less than *n*_*size*_ training samples is not partitioned any further.

### Forest Prediction

Using the randomized feature selection process, we fit the tree based on the bootstrap sample {(**X**_1_, *Y*_1_), …, (**X**_*n*_, *Y_n_*)} generated from the training data.

Let us consider the prediction based on a test sample **x** for the tree Θ. Let *η*(**x**,**Θ**) be the partition containing **x**, the tree response takes the form [15–17]:

$$y\left(x,\Theta \right)=\sum_{i=1}^{n}w_{i}\left(x,\Theta \right)y\left(i\right)$$ (3)

where the weights *w*_*i*_(**x**, Θ) are given by

$$w_{i}\left(x,\Theta \right)=\frac{1_{\left\{x_{tr}\left(i\right)\in \eta \left(x,\Theta \right)\right\}}}{\#\{r:x_{tr}\left(i\right)\in \eta \left(x_{tr}\left(r\right),\Theta \right)\}}$$ (4)

Let the *T* trees of the Random forest be denoted by Θ_1_, ⋯, Θ_*T*_ and let *w*_*i*_(**x**) denote the average weights over the forest i.e.

$$w_{i}\left(x\right)=\frac{1}{T}\sum_{j=1}^{T}w_{i}\left(x,\Theta_{j}\right).$$ (5)

The Random Forest prediction for the test sample **x** is then given by

$$\bar{y}\left(x\right)=\sum_{i=1}^{n}w_{i}\left(x\right)y\left(i\right)$$ (6)

### Multivariate Random Forest (MRF)

Let us now consider the multiple response scenario with output *y*(*i*, *k*)(*i* = 1, ⋯, *n*;*k* = 1, ⋯, *r*). The primary difference between MRF and RF is in generation of the trees with different node costs *D*_*m*_(*η*) and *D*(*η*) [13].

The node cost *D*(*η*_*P*_) = ∑_*i* ∈ *η*_*P*__(*y*(*i*) − *μ*(*ψ*_*P*_))^2^ for the univariate case is the sum of squares of the differences between the output response and the mean output response for the node. For multivariate case, we would like to use a multivariate node cost that calculates the difference between a sample point and the multivariate mean distribution. One possible measure is the sum of the squares of Mahalanobis Distances [18] as shown next:

$$D_{m}\left(\eta_{P}\right)=\sum_{i\in \eta_{P}}\left(y\left(i\right)-\mu \left(\eta_{P}\right)\right)\Lambda^{-1}{\left(y\left(i\right)-\mu \left(\eta_{P}\right)\right)}^{T}$$ (7)

where Λ is the covariance matrix, **y**(*i*) is the row vector (*y*(*i*, 1), ⋯, *y*(*i*, *r*)) and **μ**(*η*_*P*_) is the row vector denoting the mean of **y**(*i*) in node *η*_*P*_. The inverse covariance matrix (Λ^−1^) is a precision matrix [19] which is helpful to test conditional dependence between multiple random variables. The Mahalanobis distance square normalizes the output responses by their standard deviations and in case of Λ being diagonal, it represents the normalized Euclidean distance. For bivariate case with covariance Λ, the node cost is increased when the deviations of the two output responses from the mean responses are in opposite directions.

Since, the Mahalanobis distance captures the distance of the sample point from the mean of the node along the principal component axes, it might be unable to capture nonlinear relationships that produces a closer to diagonal Covariance matrix. Thus, our objective is to introduce Copulas to capture the nonlinear multivariate structure.

### Copula Description

Copulas can represent the dependence between multiple random variables independent of the marginal distributions. A copula function [20] is used to map the joint cumulative probability distribution in terms of the marginal cumulative probability distributions. Let Ψ_1_, Ψ_2_…Ψ_*N*_ represent *N* real valued random variables uniformly distributed on [0, 1]. Copula *C*: [0, 1]^*N*^ → [0, 1] with parameter *θ* is defined as:

$$C_{\theta }\left(u_{1},u_{2}...u_{N}\right)=P\left(\Psi_{1}\leq u_{1},\Psi_{2}\leq u_{2}...\Psi_{N}\leq u_{N}\right)$$ (8)

The multivariate cumulative probability distribution *F*_*X*_(*x*_1_, *x*_2_, …*x*_*N*_) and the marginal cumulative probability distributions *F*_*i*_(*x*_*i*_) for (*i* ∈ {1, 2, …*N*} are related by Sklar’s theorem [20] as follows:

$$F_{X}\left(x_{1},x_{2},...x_{N}\right)=C\left(F_{1}\left(x_{1}\right),F_{2}\left(x_{2}\right)...F_{N}\left(x_{N}\right)\right)$$ (9)

If the marginal cumulative distributions (*F*_*i*_(*x*)) are continuous, copula *C* is unique [20].

Some copulas can be parameterized using few parameters, for instance, the clayton copula [21] for bivariate distribution is defined as follows using parameter *ξ*:

$$C\left(u_{1},u_{2};\xi \right)={\left(u_{1}^{-\xi }+u_{2}^{-\xi }-1\right)}^{-1/\xi }\;;\xi \in \left(0,\infty \right)$$ (10)

Similarly, the copula characterizing two independent variables will have the form *C*(*u*_1_, *u*_2_) = *u*_1_ *u*_2_. Some other common forms of parameterized copulas include Gaussian Copula [22], Frank Copula [23], student’s t-copula [24] and Gumbel copula [25]. However, the standard forms of parameterized copulas may not capture all forms of relationships. We can consider the use of empirical copulas that are estimated directly from the cumulative multivariate distribution. Note that the calculation of empirical copulas will have higher computational complexity than parameterized copulas but they can capture a broad range of relationships. We utilize empirical copulas to represent our multivariate structures.

### Node Split Criteria using Copula

As described earlier, the regression tree generation process involves partition of a node into two branches based on optimizing a cost criterion. The node cost for univariate regression trees is given by Eq 1 and the node cost for multivariate regression trees utilizing Mahalanobis distance is shown in Eq 7. The feature and threshold that results in maximum cost reduction for that node is selected for splitting.

We next discuss the design of node cost function based on copulas to capture the output dependencies. We expect that the dependency structure among the samples in a node should be similar to the dependency structure observed in the original training data. Consider the node *η*_*P*_ with *N*_*P*_ samples and let Ψ denote the integral of the difference in the empirical copulas observed at node *η*_*P*_ and the root node (this is same as the empirical copula for the training data). We design the node cost *D*_*C*_(*η*_*P*_) for a copula based multivariate regression tree as follows:

$$D_{C}\left(\eta_{P}\right)=D_{1}+\alpha D_{2}\;\text{where}$$ (11)

$$\begin{array}{l} D_{1}=6N_{P}\Psi \;\text{and}\\ D_{2}=\sum_{j=1}^{r}\frac{\sum_{i\in \eta_{P,j}}{\left[y\left(i,j\right)-\mu \left(\eta_{P,j}\right)\right]}^{2}}{\sigma_{j}^{2}} \end{array}$$ (12)

where *α* denotes a scaling factor determining the relative weight of the two components of the node cost, *η*_*P*, *j*_ for *j* ∈ {1, ⋯, *r*} denotes the set of *j*th output responses at node *η*_*P*_ and $\sigma_{j}^{2}$ for *j* ∈ {1, ⋯, *r*} denotes the variance of the *j*th output response at root node.

We next present the motivation for selecting the weight 6 for the integral of copula distance along with approaches to select the scaling factor *α*. For maintaining *D*_1_ and *D*_2_ in the same range, we analyzed the range of Ψ as compared to *D*_2_.

Hereafter, the MRF approach that uses copula based node splitting criteria (based on Eq 11) will be termed as *CMRF* and the MRF approach using covaraince based node splitting criteria (based on Eq 7) will be termed as *VMRF*.

### Analyzing integral of differences in copulas

We first analyze the upperbound on the integral of the difference between two bivariate copulas and subsequently explore further multivariate copulas. Based on Frechet-Hoeffding bounds [26], any bivariate copula *C*(*u*, *v*) is bounded by the following:

$$C_{L}\left(u,v\right)=max\left[u+v-1,0\right]\leq C\left(u,v\right)\leq min\left[u,v\right]=C_{U}\left(u,v\right)$$

Thus for any two copulas *C*_1_(*u*, *v*) and *C*_2_(*u*, *v*), we have

$$|C_{1}\left(u,v\right)-C_{2}\left(u,v\right)|\leq C_{U}\left(u,v\right)-C_{L}\left(u,v\right)\;\forall u,v\in \left[0\;1\right]$$

Consequently,

$$\begin{array}{ll} \int_{v=0}^{1}\int_{u=0}^{1}|C_{1}(u,v)-C_{2}(u,v)|\text{d}u\text{d}v\leq & \int_{v=0}^{1}\int_{u=0}^{1}[C_{U}(u,v)\\ & -C_{L}(u,v)]\text{d}u\text{d}v \end{array}$$

Using the two diagonals in the unit square (*u* = *v* and *u* + *v* = 1), we can divide the unit square into four triangles where the values of *C*_*U*_(*u*, *v*) and *C*_*L*_(*u*, *v*) are simple functions of *u* and *v*. For region 1, we have *u* > *v* and *u* + *v* > 1 and

$$C_{U}\left(u,v\right)-C_{L}\left(u,v\right)=v-\left(u+v-1\right)=1-u$$

For region 2, we have *u* > *v* and *u* + *v* ≤ 1 and

$$C_{U}\left(u,v\right)-C_{L}\left(u,v\right)=v-0=v$$

Similarly for region 3, we have *u* ≤ *v* and *u* + *v* ≤ 1 and

$$C_{U}\left(u,v\right)-C_{L}\left(u,v\right)=u-0=u$$

And for region 4, we have *u* ≤ *v* and *u* + *v* > 1 and

$$C_{U}\left(u,v\right)-C_{L}\left(u,v\right)=u-\left(u+v-1\right)=1-v$$

The integral over region 1 is as follows:

$$\begin{array}{l} {\;\int \int \;}_{Region1}\left(C_{U}\left(u,v\right)-C_{L}\left(u,v\right)\right)\;du\;dv\\ =\int_{v=0.5}^{1}\int_{u=v}^{1}\left(1-u\right)dudv+\int_{v=0}^{0.5}\int_{u=1-v}^{1}\left(1-u\right)dudv=1/24 \end{array}$$

We can likewise show that the value of the integral for each of the there other regions is $\frac{1}{24}$. Thus $\int_{v=0}^{1}\int_{u=0}^{1}\left(C_{U}\left(u,v\right)-C_{L}\left(u,v\right)\right)dudv=1/6$ Thus, the upper bound on the surface integral of the difference between any two bivariate copulas is 1/6. Similarly, if we consider the independent copula *C*_*I*_(*u*, *v*) = *uv*, then we can show that $\int_{v=0}^{1}\int_{u=0}^{1}\left(C_{U}\left(u,v\right)-C_{I}\left(u,v\right)\right)dudv=1/12$. For *n* > 2, we conducted simulations to estimate the value of the integrals which is shown in Table 1.

**Table 1:** Integral of Copula Differences for different dimensions.

Dimensions	∫*C*_*U*_ − *C*_*L*_	∫*C*_*U*_ − *C*_*I*_	∫*C*_*U*_
2	$\frac{1}{6}$	$\frac{1}{12}$	$\frac{1}{3}$
3	0.2093	0.1255	0.252
4	0.197	0.1425	0.2072

Thus, since the upper bound of ∫(*C*_*U*_ − *C*_*L*_) lies in the range of 1/6 to 0.21, *D*_1_ = 6*N*_*P*_ Ψ will be upper bounded by *N*_*P*_ for a bivariate copula. If the regression tree is unable to reduce the initial variance in each output response, the value of *D*_2_ will be in the range of *rN*_*P*_ as the *j*th numerator term will be close to $N_{P}\sigma_{j}^{2}$. However, since the regression tree will likely reduce the variance in the output response at nodes further away from the root, the value of *D*_2_ will be much lower than *rN*_*P*_.

### Selection of *α*

Our previous analysis of Ψ provided a range of the integral difference between two copulas but was unable to provide a weight factor for combining *D*_1_ and *D*_2_ that is optimal in terms of predictive performance. We expect that the behavior of *D*_1_ and *D*_2_ will change significantly for different training datasets and thus we select *α* based on each training dataset. We next describe two techniques to select the weight factor *α* to achieve higher prediction accuracy.

#### Method 1: Evaluating and selecting from a set of *α*’s

This is a straightforward approach where different values of *α* (we considered 10 values of *α* spaced between 0.1 to 10) are evaluated and the one with best predictive performance selected. The original training data is sub-divided into secondary training and secondary testing samples. The secondary training samples are used to create MRF models that are used to find prediction of secondary testing samples. This process is repeated for a set of possible values of *α*. The correlation coefficient between predicted secondary testing samples and original secondary testing samples are recorded and the *α* corresponding to highest correlation coefficient is selected (*α*_*S*_). This *α*_*S*_ is then used to create MRF model using the original training samples and tested on original testing samples. For our examples, we have applied 10 fold CV on the original data and thus for each fold of training data, we may select different *α*. However, for each specific fold, *α* will be fixed for all the trees generated. The above method increases the computational complexity due to the evaluation of multiple values of *α*. We next present another approach that attempts to reduce the evaluation of numerous values of *α*.

#### Method 2: Pareto Frontier Approach to select *α*

**Fig 1:**
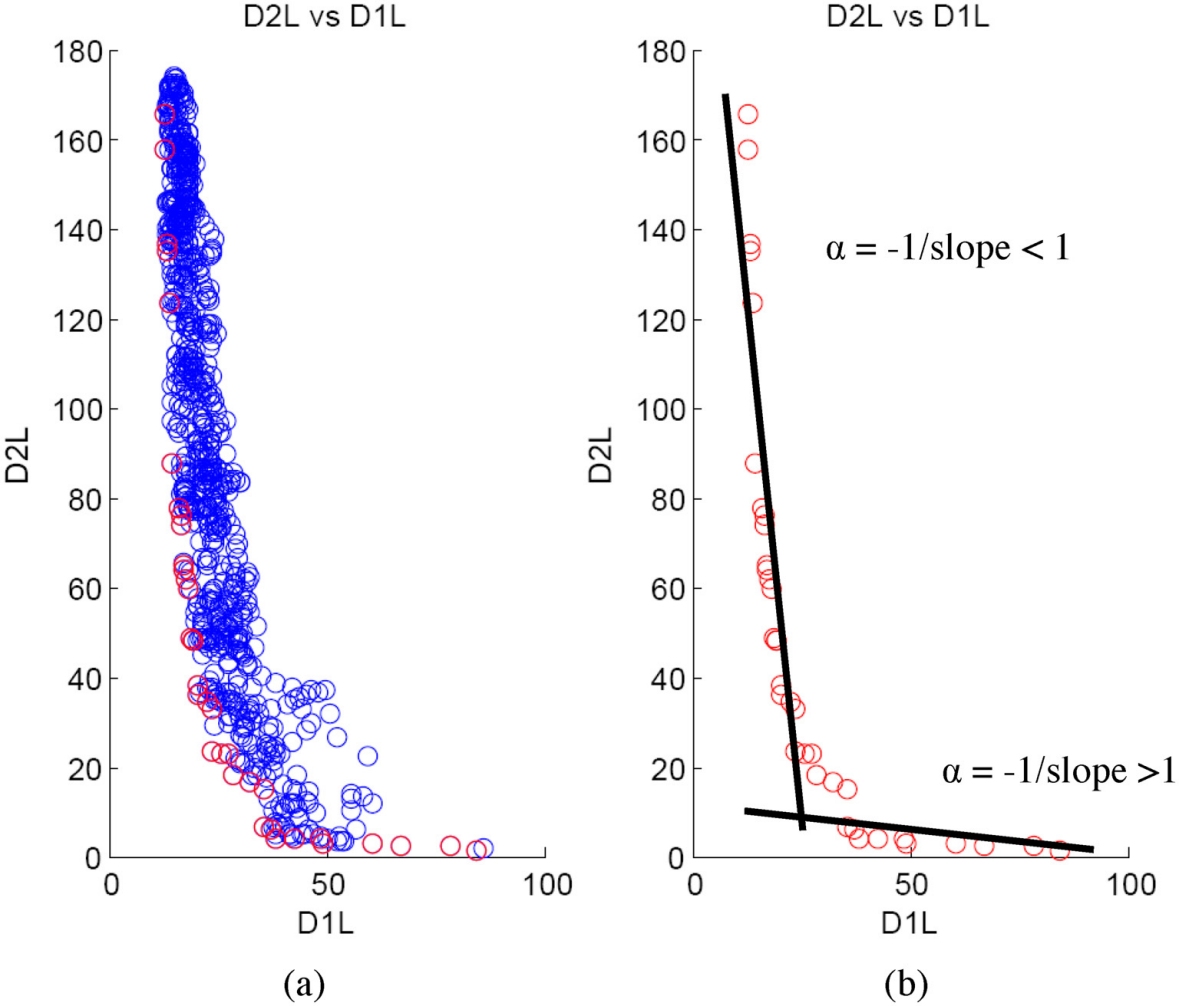
*D*_2_ vs *D*_1_ for left node. (a) shows an example Pareto frontier (red circles) for the left child node for the first split of a specific tree (the *D*_1_ and *D*_2_ values are denoted by *D*_1*L*_ and *D*_2*L*_ respectively). (b) shows that the Pareto frontier can be approximated by two straight lines: one with slope greater than 1 and another with slope less than 1.

**Fig 2:**
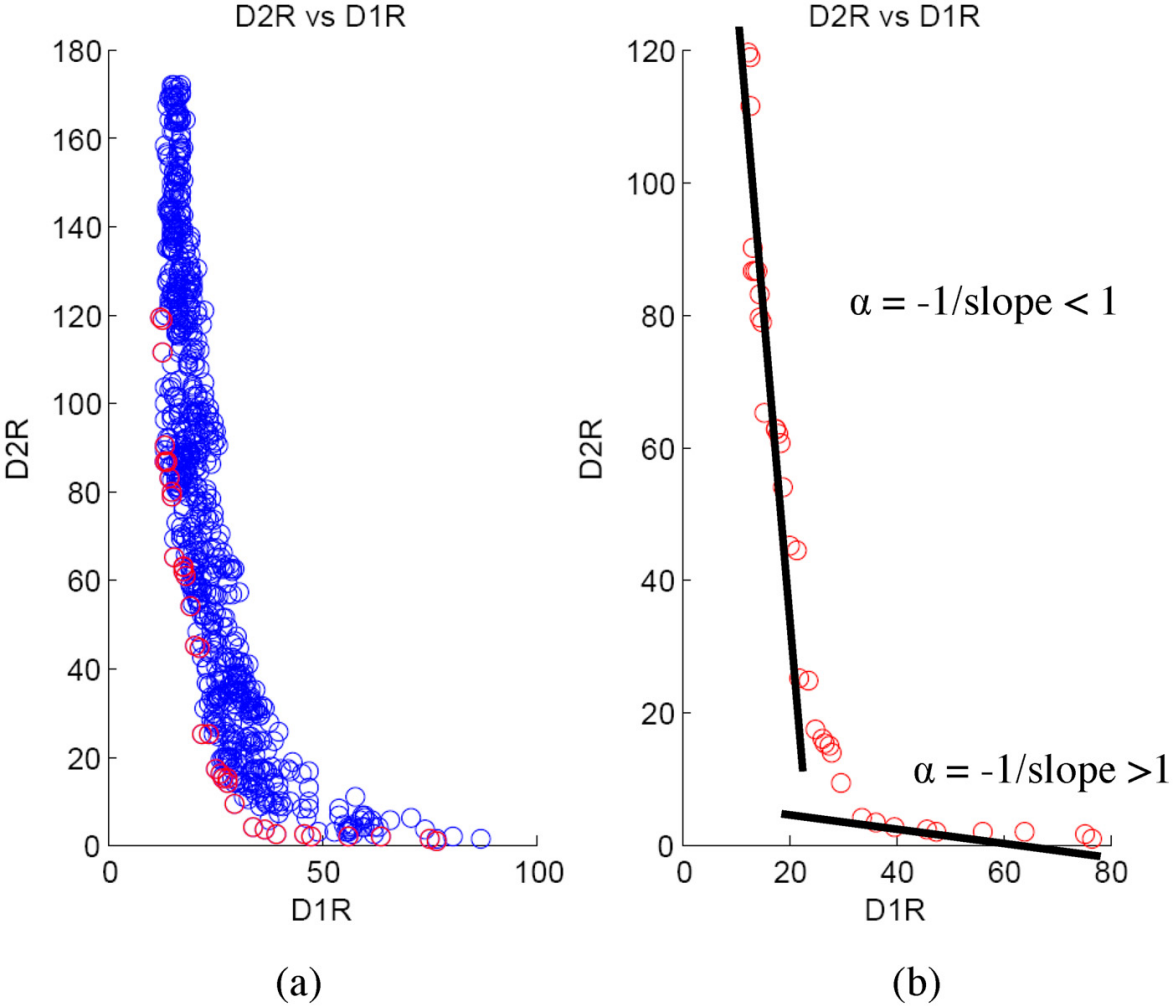
*D*_2_ vs *D*_1_ for right node. (a) shows an example Pareto frontier (red circles) for the right child node for the first split of a specific tree (the *D*_1_ and *D*_2_ values are denoted by *D*_1*R*_ and *D*_2*R*_ respectively). (b) shows that the Pareto frontier can be approximated by two straight lines: one with slope greater than 1 and another with slope less than 1.

In this approach, we consider the node cost function minimization from a multi-objective optimization problem perspective where we aim to jointly minimize both *D*_1_ and *D*_2_. From the multi-objective perspective, if we plot the *D*_2_ vs *D*_1_ for all possible feature and threshold combinations for a specific node (if we have *n* samples at a specific node and *m* features, the number of partitions to be evaluated is *mn*), we should select a feature and threshold combination that lies in the Pareto frontier. In other words, we look for solutions that are not dominated by any other solution: for instance if the *D*_1_ and *D*_2_ values for *w* different feature and threshold combinations are denoted by {*ϵ*_1_(*i*), *ϵ*_2_(*i*)} for *i* ∈ {1, ⋯, *w*}, a combination *i* is considered dominated by *j* if either (a) *ϵ*_1_(*i*)>*ϵ*_1_(*j*) and *ϵ*_2_(*i*)≥*ϵ*_2_(*j*) or (b) *ϵ*_1_(*i*)≥*ϵ*_1_(*j*) and *ϵ*_2_(*i*)>*ϵ*_2_(*j*) is valid. The feature and threshold combinations that are not dominated by any of the other *w* − 1 combinations form the Pareto Frontier. For instance, Fig 1(a) shows an example Pareto frontier (red circles) for the left child node for the first split of a specific tree (the *D*_1_ and *D*_2_ values are denoted by *D*_1*L*_ and *D*_2*L*_ respectively) generated from a synthetic example described in next section. Similarly, Fig 2(a) shows the Pareto frontier (red circles) for the right child node for the first split of a specific tree (the *D*_1_ and *D*_2_ values are denoted by *D*_1*R*_ and *D*_2*R*_ respectively).

Our idea is to approximate the Pareto frontier using straight lines and utilize the slope of the lines to design *α*. Figs 1(b) and 2(b) shows that the Pareto frontier can be approximated by two straight lines: one with slope greater than 1 and another with slope less than 1. Consequently, the value of *α* can be approximated by the following equation.

**Fig 3:**
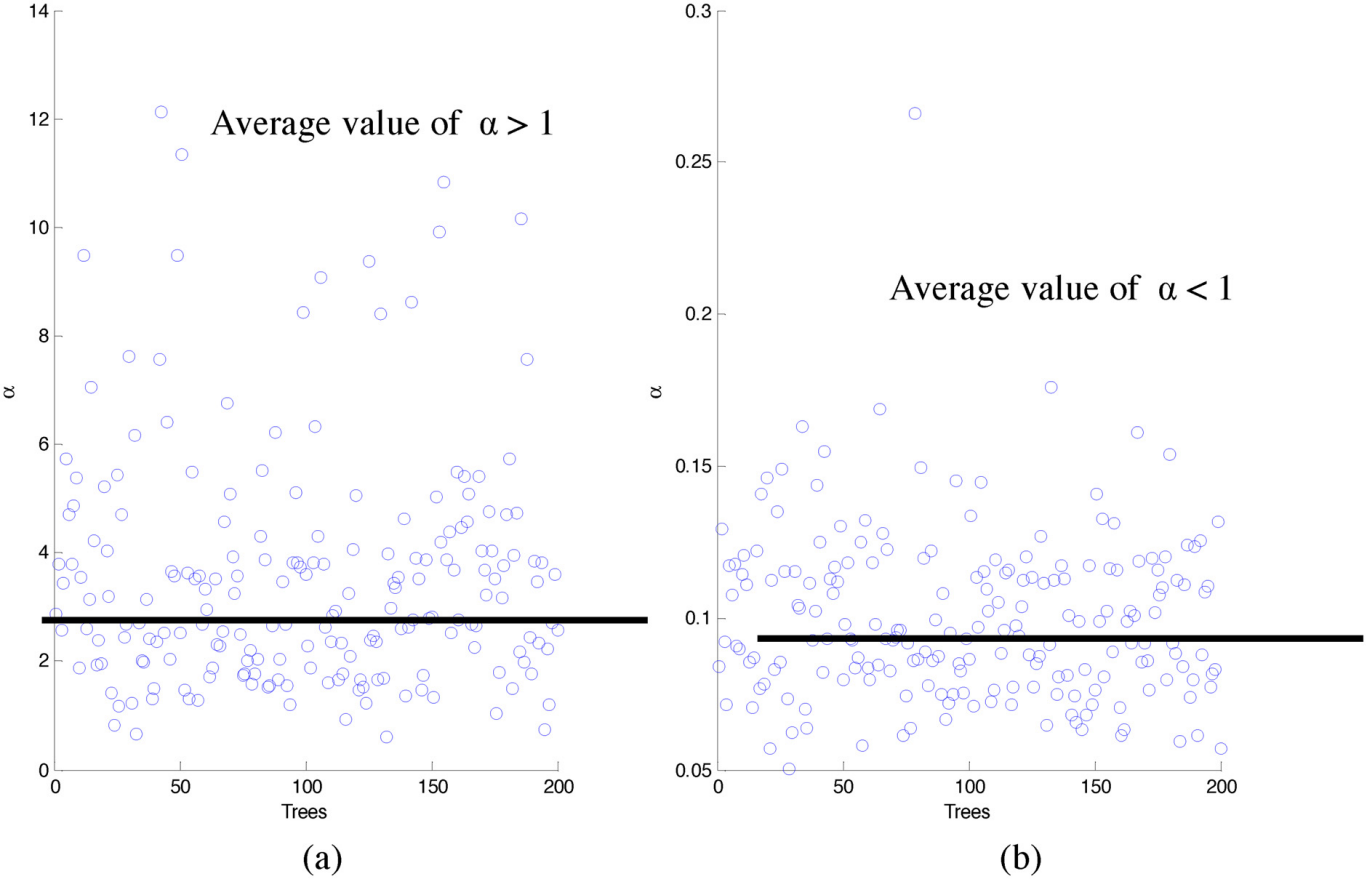
Scatter plot of *α*’s across the trees. (a) and (b) are scatter-plots for the first split of all the trees for *α* > 1 and *α* < 1 respectively.

α=-1/ρ

where *ρ* denotes the slope of the straight line fitted to the Pareto frontier. Thus, we have 2 possible values of *α* from the very first split of a specific tree. If we prepare scatter-plots for the first split of all the trees for *α* > 1 and *α* < 1, we arrive at plots similar to Fig 3(a) and 3(b) respectively. In this method, we calculate the predictive performance of the two average *α*’s and select the one with the best performance to design the overall forest.

### An example to illustrate the appropriateness of Copula based node cost for design of Multivariate Regression Trees

We have observed that multivariate random forests incorporating covariance (Mahalanobis distance square) between output responses is more suitable for predicting output responses with linear relationships as compared to output responses with non-linear relationships. Copula presents a methodology to capture the non-linear dependence relationships between multiple variables; and we anticipate that copula will be suitable for predicting output drug responses with non-linear relationships between them. We next present a synthetic example with non-linear relationship to investigate the performance of the proposed approach as compared to covariance based MRF design.

We consider a 50 × 10 input data matrix (50 samples and 10 features) denoted by **X** that was created randomly from a normal distribution N(0,1). We next generated two output responses **Y**_1_ and **Y**_2_ based on functions of the input features. Let column vectors **x**_*i*_(*i* = 1, 2, ⋯, 10) denote the 10 features and the output responses **Y**_1_ and **Y**_2_ are defined as follows:

$$Y_{1}=2x_{1}+5x_{2}-1.5x_{3}+x_{4}$$ (13)

$$Y_{2}={\left(Y_{1}-E\left(Y_{1}\right)\right)}^{2}$$ (14)

Note that the output responses are dependent on only 4 features out of the 10 possible input features. Based on the relative weights, **x**_2_ is the most weighted feature and should play a critical role while growing the trees at the beginning. Note that *Y*_1_ and *Y*_2_ has a quadratic relationship.

We consider two multivariate regression trees trained on the same input **X** and same output responses [**Y**_1_,**Y**_2_] but different node splitting criterion. The regression trees denoted by *Tree*{**V**, [**Y**_1_,**Y**_2_]} and *Tree*{**C**, [**Y**_1_,**Y**_2_]} are inferred using the covariance (Eq 7) and copula (Eq 11) based node cost functions respectively. For this example, all the features were considered at each node i.e. *m* = *M* and randomly chosen 80% of 50 samples with bootstrapping were used for generating the regression trees.

**Fig 4:**
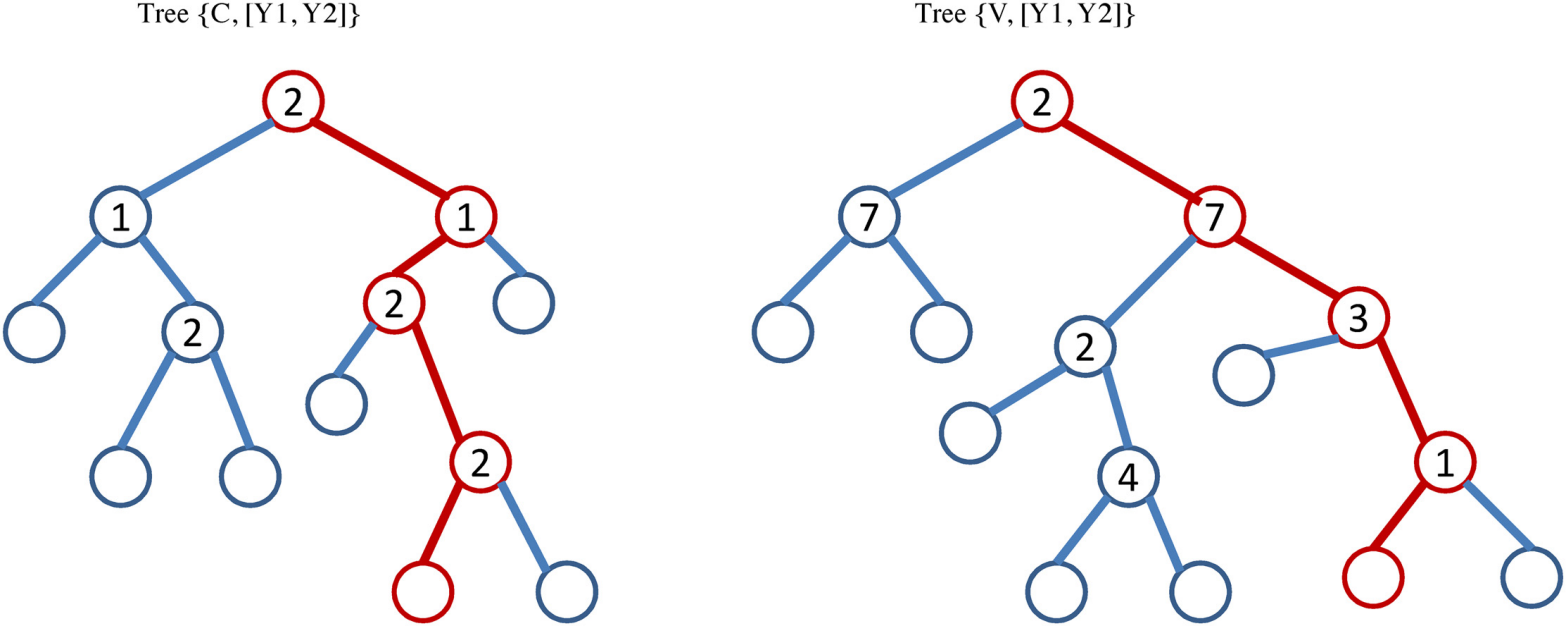
Two multivariate regression trees trained on the same input X and same output responses [Y_1_,Y_2_] but the node cost criteria being copula based (*Tree*{C, [Y_1_,Y_2_]}) and covariance based (*Tree*{V, [Y_1_,Y_2_]}) respectively. The empty circles represent leaf nodes and the circles enclosing a number signifies a split node; the number inside the circle indicates the featured selected on that node for splitting.

Fig 4 shows two multivariate regression trees generated using copula (Eq 11) and covariance (Eq 7) based node cost functions. Fig 4 illustrates that the splitting process for each tree is dependent on different features at each node, which eventually leads to two totally dissimilar trees. The empty circles denote leaf nodes; the circles enclosing a number signify a split node and the number inside the circle indicate the featured selected on that node for splitting.

We expect that the regression tree generation based on copula as compared to covariance will be able to better capture the non-linear relationship between *Y*_1_ and *Y*_2_. Fig 4 demonstrates that the copula based *Tree*{**C**, [**Y**_1_,**Y**_2_]} has selected the most significant features (features 2 and 1 that have the highest weights during generation of *Y*_1_ and *Y*_2_) while generating the multivariate regression tree. On the hand, the covariance based tree *Tree*{**V**, [**Y**_1_,**Y**_2_]} trained on the same data selected a spurious feature 7 which was not involved in the generation of either *Y*_1_ or *Y*_2_.

**Fig 5:**
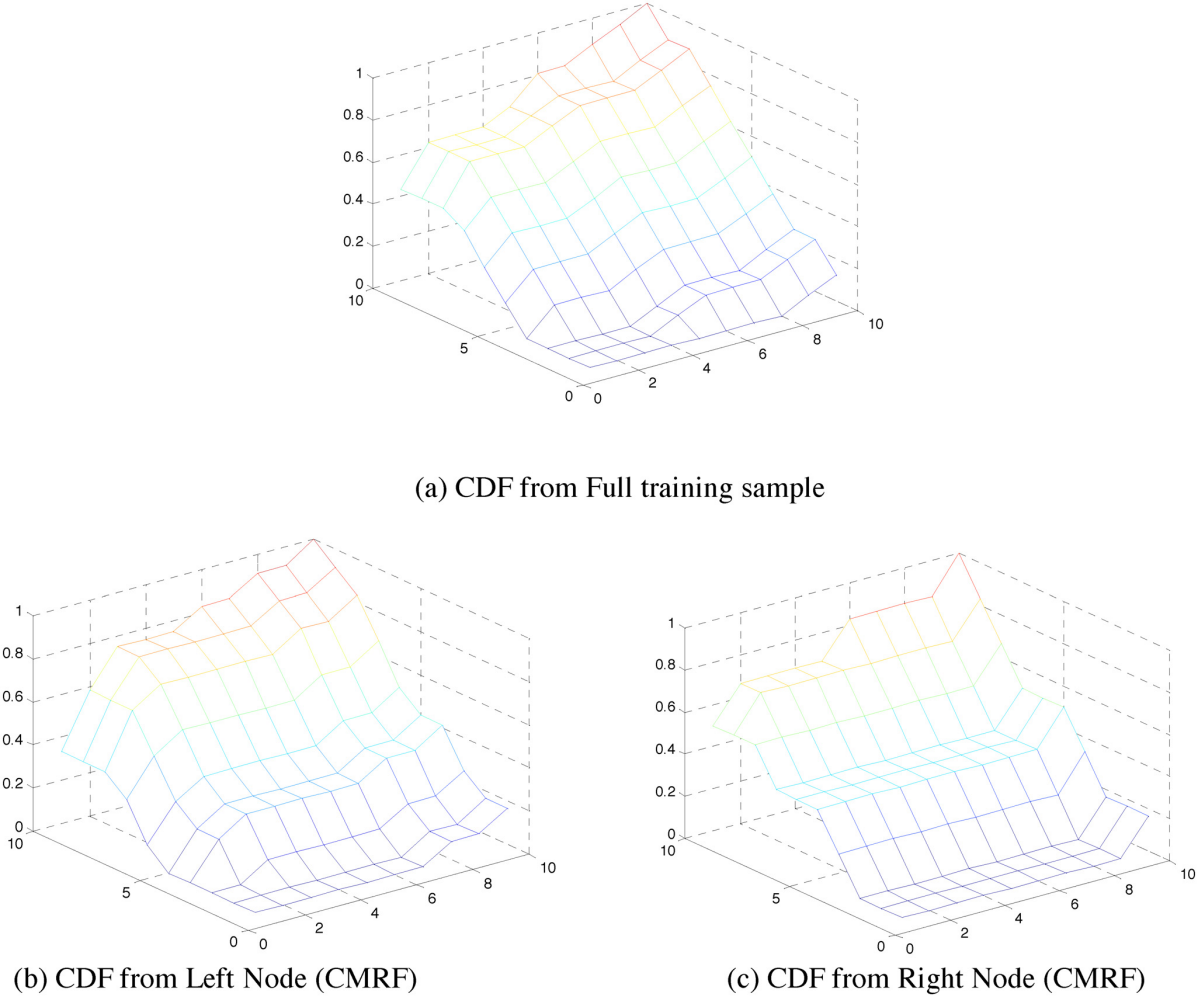
CDF created from left and right child node for a single split using CMRF. It is compared visually with the original CDF created from the training samples.

**Fig 6:**
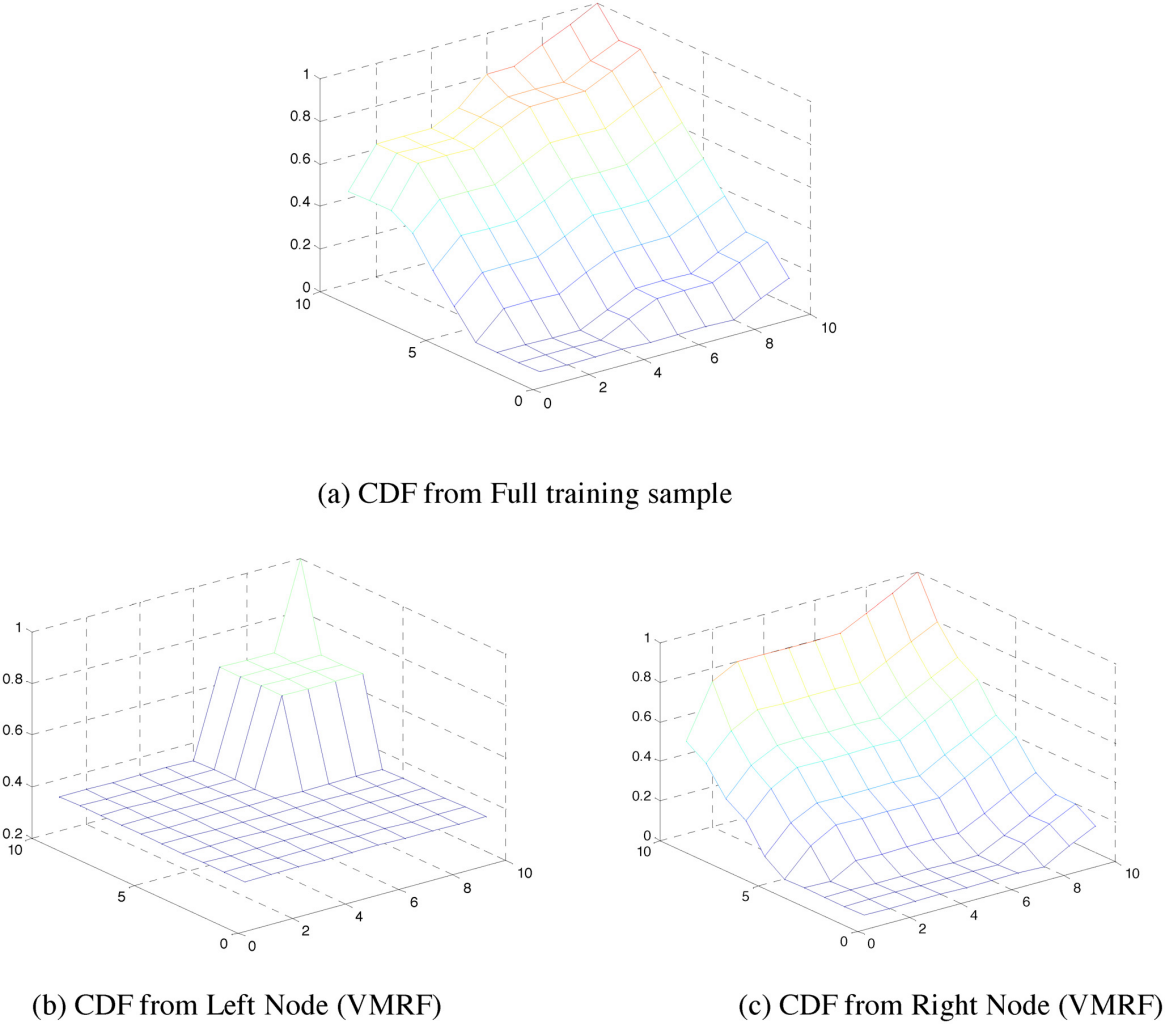
CDF created from left and right child node for a single split using VMRF. It is compared visually with the original CDF created from the training samples.

To visually compare the multivariate structure during regression tree splits, we plotted the cumulative distribution functions (CDFs) for the original data and after splitting using copula and covariance based node cost functions. Fig 5 shows the original CDF and the CDFs at the left and right child nodes when the node split is based on Eq 11 (CMRF). Likewise, Fig 6 shows the original CDF and the CDFs at the left and right child nodes when the node split is based on Eq 7 (VMRF). We observe that the node split using copula based node cost better maintains the CDF observed in the original data (Fig 5(b) and 5(c) are similar to (a)) as compared to the split using covariance based node cost (Fig 6(b) is significantly different from (a)).

#### Variable Importance Measure (VIM)

In this section, we consider the issue of feature selection for MRFs. We would like to generate and compare the Variable Importance Measure (VIM) for CMRF and VMRF. We expect that CMRF will have higher feature scores for the significant features as compared to VMRF. Typical variable importance measure for random forest considers the frequency of feature selection, out of bag error or permutation measures [27]. We consider the basic approach of calculating the number of times each feature gets selected and the VIM for each forest will be the sum of these frequencies across all trees normalized to the range between 0 and 1. Based on the synthetic data, we generated 100 Multivariate Regression Trees using CMRF (with fixed *α*) and VMRF with output responses *Y*_1_ and *Y*_2_ and generated the variable importance of the 10 input features. The normalized variable importance scores reported in Table 2 illustrate that the top four features selected by CMRF (*X*_2_, *X*_1_, *X*_3_, *X*_4_) are the same as the four features that were used to generate *Y*_1_ and *Y*_2_ using Eqs 13 and 14 respectively. Furthermore, the ordering of the scores *VIM*(*X*_2_)>*VIM*(*X*_1_)>*VIM*(*X*_3_)>*VIM*(*X*_4_) is same as the ordering of the absolute weights of the four features in generation of *Y*_1_ where *X*_2_ has the largest weight followed by *X*_1_, *X*_3_ and *X*_4_. On the other hand, the top four features selected by *VMRF* *X*_2_, *X*_1_, *X*_3_, *X*_6_ fails to pick *X*_4_ and includes a spurious feature *X*_6_ that was not involved in the generation of output response *Y*_1_. Thus, the example supports that copula based MRF might be better suitable to select top features as compared to covariance based MRF.

**Table 2:** Variable importance measure calculated using *CMRF*_*Y*_1_, *Y*_2__ and *VMRF*_*Y*_1_, *Y*_2__.

*Feature*	*X*_1_	*X*_2_	*X*_3_	*X*_4_	*X*_5_	*X*_6_	*X*_7_	*X*_8_	*X*_9_	*X*_10_
*CMRF*_*Y*_1_, *Y*_2__	0.2440	0.3720	0.0952	0.0744	0.0179	0.0625	0.0238	0.0417	0.0327	0.0357
*VMRF*_*Y*_1_, *Y*_2__	0.2340	0.3700	0.0836	0.0529	0.0056	0.0729	0.0418	0.0418	0.0474	0.0501

## Results

For analyzing the prediction capabilities of our framework, we considered two different datasets: GDSC and CCLE. Both include genomic characterization of numerous cell lines and different drug responses for each cell line. For the current analysis, we consider the gene expression data as the genomic characterization information for both datasets. Area under the Curves (AUC) is used as representation of drug responses for both GDSC and CCLE. Both datasets are high dimensional in the number of features (gene expressions). For all performance comparison results presented in this article, a prior feature selection method (RELIEFF [28]) is applied to reduce the number of features to be used for training. A performance comparison of random forest approaches with and without the application of prior feature selection is shown for GDSC dataset in Table A in S1 File.

For performance comparison purposes, we report results of Copula based MRF (CMRF) along with univariate RF (denoted by RF), Covariance based MRF (VMRF) and Kernelized Bayesian Multitask Learning (KBMTL) [11] approaches. KBMTL is Bayesian formulation that combines kernel based non-linear dimensionality reduction and regression in a *multitask learning* framework, that tries to solve distinct but related tasks jointly to improve overall generalization performance. We have implemented KBMTL using the algorithmic code provided in [11]. Based on the parameters used in [11], we have considered 200 iterations and gamma prior values (both *α* and *β*) of 1. Subspace dimensionality has been considered to be 20 and the standard deviation of hidden representations and weight parameters are selected to be the default 0.1 and 1 respectively.

### Results on GDSC Dataset

The GDSC gene expression and drug sensitivity dataset was downloaded from Cancerrxgene.org [29]. The dataset has 789 cell lines with gene expression data and 714 cell lines with drug response data. We considered the intersection of cell lines that had both drug response and gene expression data.

For our experiments, we consider four sets of drug pairs where three of them have common primary targets and the remaining pair has no common target. We expect that the drug pairs with common primary targets will have some form of relationship among their sensitivities and CMRF should perform better than VMRF and both should perform better than RF approach. On the other hand, the drug pair without any common targets is expected to have minimal relationship among the drug sensitivities and thus RF is expected to outperform CMRF and VMRF. We also present results on 3 drug set and 138 drug set for GDSC as Tables C, D and E in S1 File.

Initially each cell line has 22277 features (probeset) as gene expressions. We have reduced it to 500 for each drug response using RELIEFF [28] and used a union of the 500 features in each of the four sets of drugs.

The first selected set *S*_*C*2_ consisting of {*Erlotinib*, *Lapatinib*} has common target *EGFR*[30–32]. The second set *S*_*C*3_ consisting of {*AZD-0530*, *TAE-684*} has common target *ABL1*[32]. The third set *S*_*C*1_ was {*AZD6244*, *PD-0325901*} with common target *MEK*[32–34]. The fourth set *S*_*U*_ consisting of {*17-AAG, Erlotinib*} has no common target.

As mentioned earlier, each drug has some missing responses across the 714 cell lines. The drug sets *S*_*C*1_, *S*_*C*2_, *S*_*C*3_ and *S*_*U*_ have drug responses in both drugs for 316, 349, 645 and 300 cell lines respectively. To report our results, we compared 5 fold cross-validated Pearson correlation coefficients, Mean Absolute Error (MAE) and Normalized Root Mean Square Error (NRMSE) between predicted and experimental responses for RF, VMRF, CMRF and KBMTL. NRMSE of drug *m* can be calculated as [11]:

$$NRMSE_{m}=\sqrt{\frac{{\left(y_{m}-{\hat{y}}_{m}\right)}^{T}\;\left(y_{m}-{\hat{y}}_{m}\right)}{{\left(y_{m}-1\cdot E\left(y_{m}\right)\right)}^{T}\;\left(y_{m}-1\cdot E\left(y_{m}\right)\right)}}$$ (15)

where *y*_*m*_ and ${\hat{y}}_{m}$ denote the vector of actual and predicted drug sensitivities respectively and **E**(*y*_*m*_) denote mean of vector *y*_*m*_. For both VMRF and CMRF, we set the minimum size of samples in each leaf to *n*_*size*_ = 5, the number of trees in the forest to *T* = 150 and the splitting in each node considers *m* = 10 random features.

The correlation coefficients using 5 fold cross validation error estimation are illustrated for each drug set in Table 3. The corresponding MAE and NRMSE behaviors are illustrated in Table 4.

**Table 3:** 5 fold CV results for GDSC Dataset drug sensitivity prediction for four drug sets in the form of correlation coefficients. *VMRF*, *CMRF* represent Multivariate Random Forest using Covariance and Copula respectively. *KBMTL* represents Kernelized Bayesian multitask learning (Parameters considered are 200 iterations, *α* = *β* = 1 and subspace dimensionality = 20).

			Correlation Co-efficients			
Drug Set	Common Target	Drug Name	*RF*	*VMRF*	*CMRF*	*KBMTL*
*S*_*C*1_	EGFR	Erlotinib	0.5156	0.5193	0.5301	0.2500
		Lapatinib	0.5544	0.5742	0.5699	0.1132
*S*_*C*2_	ABL1	AZD-0530	0.3553	0.3810	0.3990	0.3181
		TAE-684	0.4060	0.4100	0.4338	0.2420
*S*_*C*3_	MEK	AZD6244	0.4625	0.4508	0.4590	0.0950
		PD-0325901	0.5890	0.6022	0.6016	0.3236
*S*_*U*_	None	17-AAG	0.6304	0.6244	0.6167	0.4375
		Erlotinib	0.5859	0.5906	0.5708	0.4081

**Table 4:** 5 fold CV results for GDSC Dataset drug sensitivity prediction for four drug sets in the form of MAE and NRMSE for RF, VMRF, CMRF and KBMTL approaches.

			MAE				NRMSE			
Drug Set	Common Target	Drug Name	*RF*	*VMRF*	*CMRF*	*KBMTL*	*RF*	*VMRF*	*CMRF*	*KBMTL*
*S*_*C*1_	EGFR	Erlotinib	0.0319	0.0322	0.0314	0.0503	0.8733	0.8749	0.8719	1.3365
		Lapatinib	0.0292	0.0294	0.0286	0.0488	0.8516	0.8459	0.8486	1.3538
*S*_*C*2_	ABL1	AZD-0530	0.0446	0.0448	0.0442	0.0613	0.9407	0.9378	0.9291	1.2344
		TAE-684	0.0829	0.0829	0.0821	0.1159	0.9285	0.9299	0.9195	1.3698
*S*_*C*3_	MEK	AZD6244	0.0584	0.0590	0.0584	0.1138	0.8949	0.9034	0.8962	1.8016
		PD-0325901	0.0723	0.0727	0.0717	0.1199	0.8263	0.8230	0.8193	1.4028
*S*_*U*_	None	17-AAG	0.0584	0.0590	0.0584	0.1198	0.7840	0.7894	0.7955	1.1624
		Erlotinib	0.0723	0.0727	0.0717	0.0410	0.8335	0.8441	0.8505	1.1013

**Fig 7:**
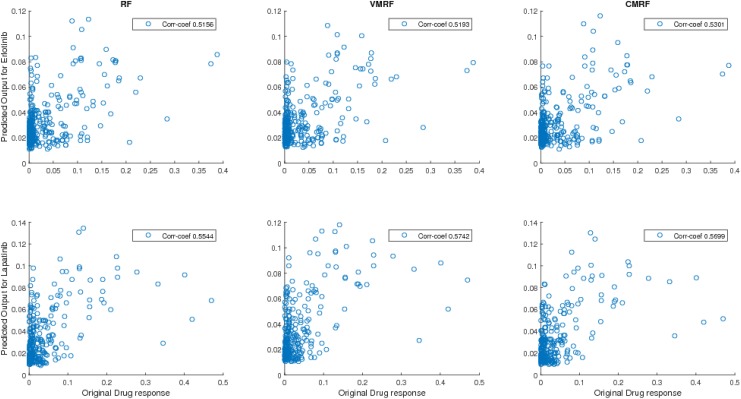
Scatter plots of predicted response vs original response for Erlotinib and Lapatinib (GDSC). Here corr-coef stands for correlation coefficient between predicted response and output response.

For CMRF, results with scaling factor *α* selected using *Method-1* discussed earlier has been used. The robustness analysis of *α* using synthetic data is conducted using *Method-2* and is shown as Tables H and I in S1 File. Table 3 shows that CMRF outperformed (in terms of correlation coefficients) VMRF, RF and KBMTL for the related drug pairs *S*_*C*1_, *S*_*C*2_, *S*_*C*3_ whereas CMRF is outperformed by the other approaches for the unrelated drug pair *S*_*U*_. Table 4 shows that *CMRF* outperforms VMRF, KBMTL and RF in terms of average NRMSE for the related pairs of drugs *S*_*c*_1__, *S*_*c*_2__ and *S*_*c*_3__. For the unrelated pair *S*_*U*_, univariate RF outperforms the multivariate approaches for both average correlation coefficients and NRMSE. The scatter plots of predicted response vs original response for drugset *S*_*C*1_ using RF, VMRF, CMRF are shown in Fig 7.

### Results on CCLE Dataset

The CCLE [35] database includes genomic characterization for 1037 cell lines and drug responses over 24 drugs for over 480 cell lines. For the purpose of predicting responses, 4 sets of drugs were selected. The first set *S*_*C*1_ = {*Erlotinib*, *Lapatinib*} has *EGFR* as a common target [30, 31], the second set *S*_*C*2_ = {*PF-02341066(Crizotinib), PHA-665752*} has *MET* as a common target [36, 37], the third set *S*_*C*3_ = {*ZD6474(Vandetanib), AZD0530(Saracatinib)*} has *EGFR* as a common target [38, 39] and the fourth set *S*_4_ = {*17-AAG, Erlotinib*} has no common target. We also include results on 4 drug set and 24 drug sets for CCLE as Tables A, F and G in S1 File.

Initially, each cell line had 18,988 features (probeset) as gene expressions. We reduced it to 500 for each drug response using RELIEFF [28] feature selection and considered a union of the 500 features in each of the four sets of drugs. We have used first 300 cell lines that have gene expression and drug responses for specific pairs of drugs. To report our results, we compared 5 fold cross-validated Pearson correlation coefficients, MAE and NRMSE between predicted and experimental responses for RF, VMRF, CMRF and KBMTL. For both VMRF and CMRF, we set the minimum size of samples in each leaf to *n*_*size*_ = 5, the number of trees in the forest to *T* = 150 and the splitting in each node considers *m* = 10 random features.

**Fig 8:**
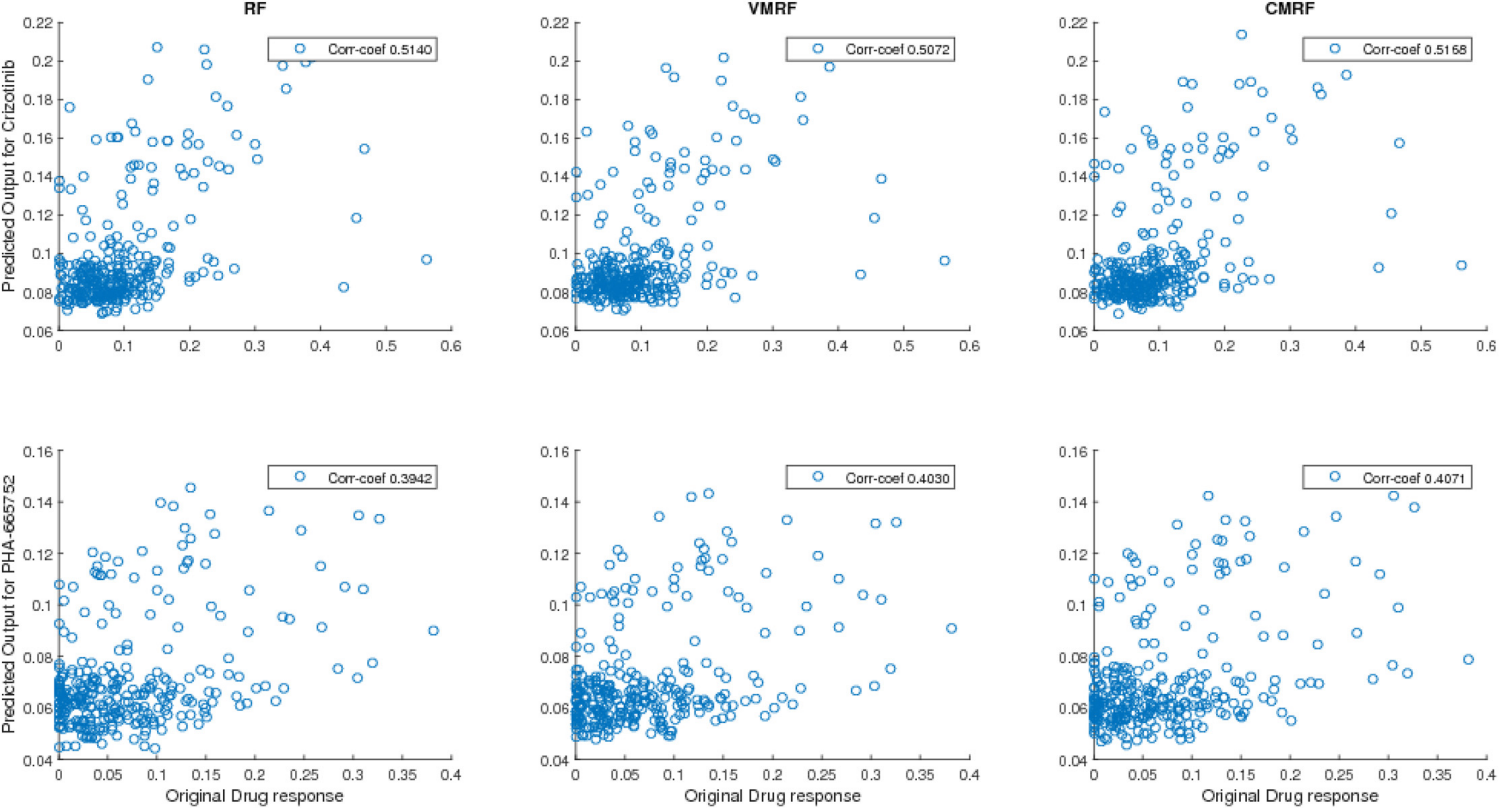
Scatter plots of predicted response vs original response for Crizotinib and PHA-665752 (CCLE). Here corr-coef stands for correlation coefficient between predicted response and output response.

The correlation coefficients using 5 fold cross validation error estimation are illustrated for each drug set in Table 5. The corresponding MAE and NRMSE behaviors are illustrated in Table 6. For CMRF, results with scaling factor *α* selected using *Method-1* discussed earlier has been used. Tables 5 and 6 shows that CMRF performed better than VMRF, KBMTL and RF in terms of correlation coefficients and NRMSE for the related drug pairs *S*_*C*1_, *S*_*C*2_, and *S*_*C*3_. When there is no relationship in the drug pair as in *S*_*U*_, univariate RF performs better than the multivariate approaches on an average. The scatter plots of predicted response vs original response for drug-set *S*_*C*2_ using RF, VMRF, CMRF are shown in Fig 8.

**Table 5:** 5 fold CV results for CCLE Dataset drug sensitivity prediction for four drug sets in the form of correlation coefficients for *RF*, *VMRF*, *CMRF* and *KBMTL*.

			Correlation Co-efficients			
Drug Set	Common Target	Drug Name	*RF*	*VMRF*	*CMRF*	*KBMTL*
*S*_*C*1_	EGFR	Erlotinib	0.3916	0.3980	0.3927	0.3457
		Lapatinib	0.4460	0.4468	0.4673	0.2609
*S*_*C*2_	MET	Crizotinib	0.4813	0.4719	0.4882	0.4519
		PHA-665752	0.3547	0.3587	0.3746	0.2250
*S*_*C*3_	EGFR	ZD-6474	0.2355	0.2535	0.2627	0.1304
		AZD-0530	0.1990	0.1844	0.1957	0.1973
*S*_*U*_	None	17-AAG	0.3620	0.3337	0.3255	0.4100
		Erlotinib	0.3818	0.3852	0.3718	0.2828

**Table 6:** 5 fold CV results for CCLE Dataset drug sensitivity prediction for four drug sets in the form of MAE and NRMSE for *RF*, *VMRF*, *CMRF* and *KBMTL*.

			MAE				NRMSE			
Drug Set	Common Target	Drug Name	*RF*	*VMRF*	*CMRF*	*KBMTL*	*RF*	*VMRF*	*CMRF*	*KBMTL*
*S*_*C*1_	EGFR	Erlotinib	0.0522	0.0520	0.0515	0.0612	0.9223	0.9210	0.9218	1.0593
		Lapatinib	0.0513	0.0520	0.0509	0.0654	0.8976	0.8977	0.8895	1.1398
*S*_*C*2_	MET	Crizotinib	0.0484	0.0483	0.0477	0.0546	0.8836	0.8921	0.8828	0.9674
		PHA-665752	0.0492	0.0496	0.0489	0.0614	0.9367	0.9367	0.9307	1.1573
*S*_*C*3_	EGFR	ZD-6474	0.0660	0.0659	0.0656	0.0876	0.9721	0.9674	0.9650	1.3037
		AZD-0530	0.0728	0.0728	0.0727	0.0866	0.9801	0.9834	0.9810	1.2188
*S*_*U*_	None	17-AAG	0.1003	0.1005	0.1008	0.0997	0.9553	0.9614	0.9644	0.9740
		Erlotinib	0.0517	0.0519	0.0520	0.0612	0.9258	0.9260	0.9311	1.0957

### Results of Variable Importance Analysis

We have examined the variable importance measure for GDSC data using VMRF and CMRF in terms of protein interaction network enrichment analysis. In this section, we will primarily provide the detailed results for *S*_*C*1_ in GDSC. To avoid any bias due to feature selection in variable importance, we consider the full set of probe set ids without application of RELIEFF for this analysis.

**Fig 9:**
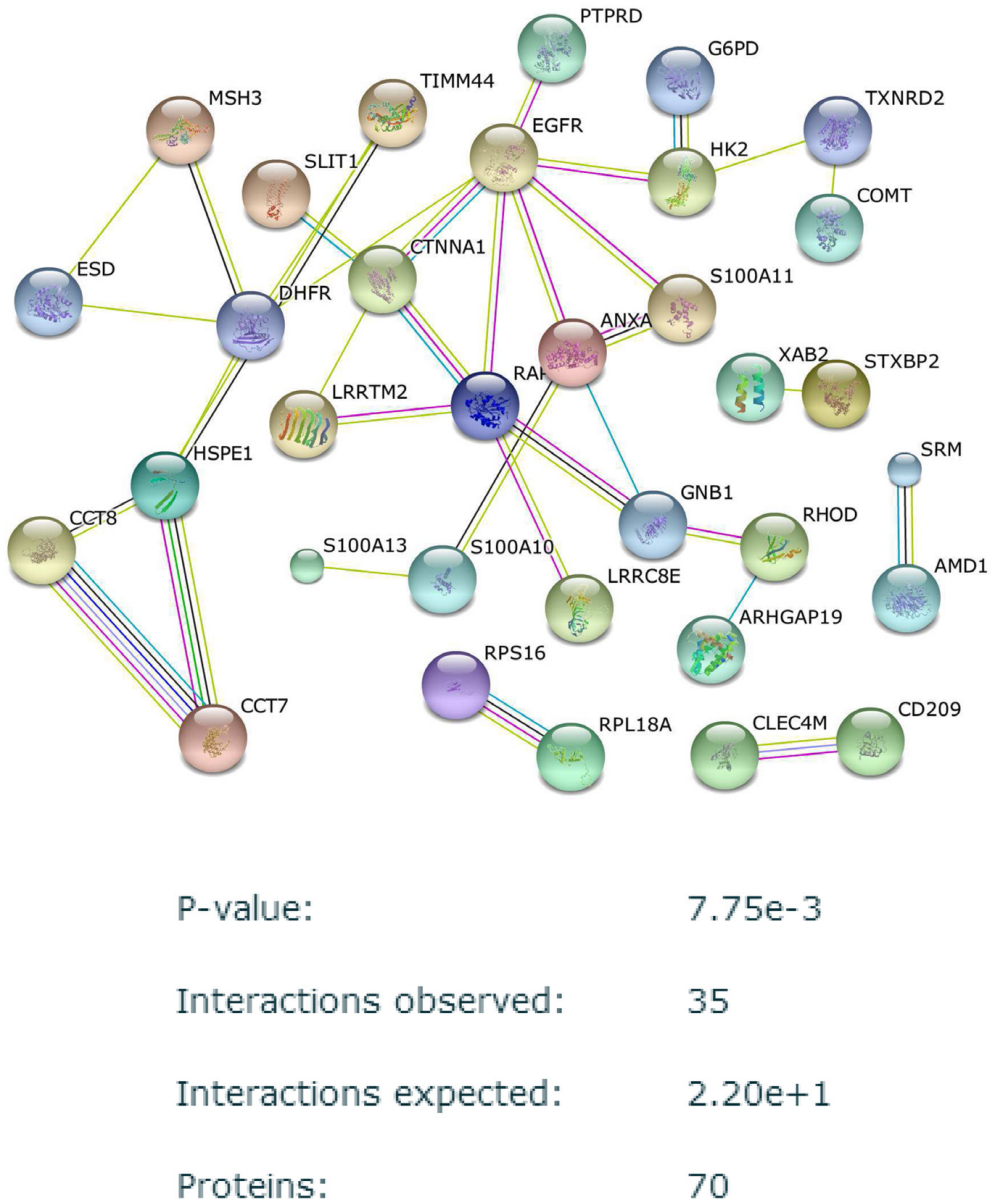
Protein-protein interaction network observed between top regulators found from CMRF in GDSC dataset *S*_*C*1_. Disconnected nodes are hidden.

**Fig 10:**
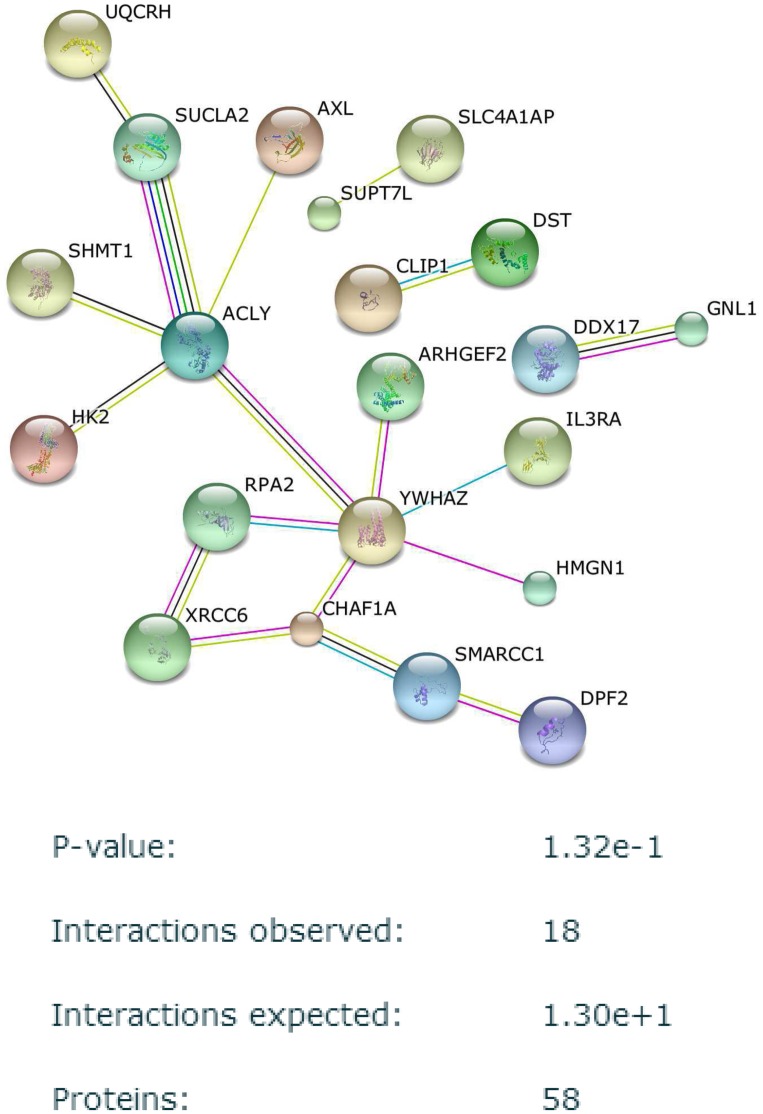
Protein-protein interaction network observed between top regulators found from VMRF in GDSC dataset *S*_*C*1_. Disconnected nodes are hidden.

In both VMRF and CMRF, the 50 top ranked probesets were generated separately. It should be noted that multiple probeset IDs can map to a single Gene Symbol of a protein. This mapping was done in Genome Medicine Database of Japan (GeMDBJ) ID conversion tool (https://gemdbj.nibio.go.jp/dgdb/ConvertOperation.do). Based on this mapping, we arrived at 58 top ranked proteins for VMRF and 70 top ranked proteins for CMRF. These proteins were provided as inputs to the string-db database (http://string-db.org/) for known protein-protein interactions. The protein-protein interaction (PPI) networks for top proteins using CMRF and VMRF are shown in Figs 9 and 10 respectively. The enrichment analysis for both the networks are shown alongside each network. We observe that the network generated using CMRF is more enriched in connectivity than the network generated using VMRF. 18 interactions with a p-value of 0.132 were observed for the VMRF PPI network whereas a total of 35 interactions with a p-value of 0.00775 were observed for the CMRF network. Moreover, the common target EGFR is picked in the top 50 targets and is well connected to other targets of CMRF whereas EGFR is not selected even in top 150 targets of VMRF.

Similarly, in drugset *S*_*C*2_ of GDSC (network not shown), there are 42 interactions with 51 proteins in CMRF and 25 interactions with 54 proteins in VMRF.

## Conclusions

In this article, we presented an approach to extend ensemble learning using regression trees to multivariate ensemble learning. We utilized the concept of copulas to represent the relationship between different drug sensitivities and incorporated them in the design of multivariate regression tree cost function. We designed the node cost function as a combination of (a) the sum of square of the differences from the mean and (b) a measure of the difference in the multivariate structure at the node compared to the original training data. The difference in the multivariate structure was captured as the integral of the absolute difference in the copulas observed at the node and the original training data. Two approaches were presented based on enumeration and Pareto frontier to design the weights of the two parts of the cost function. Utilizing synthetic and biological data, we showed that the proposed copula based approach could increase the prediction accuracy as compared to univariate random forests or multivariate random forests based on covariance based node cost. As compared to RF, the gain in the correlation coefficient between predicted and experimental values was observed in scenarios where there exists a relationship between the drug pair sensitivities. The examples were also able to illustrate that CMRF is better suited for selecting the relevant features as compared to VMRF. The proposed methodology provides a novel technique to design multivariate regression trees for scenarios where there are nonlinear relationships between output responses. The presented research can be extended in multiple directions. One such direction will involve extending the concept of maintaining the multivariate structure in the design of weights of individual trees. Another direction consists of analyzing the detailed bias and variance relationship of the proposed technique and designing confidence intervals for the predictions.

## Supporting Information

S1 File Supporting Information for Article: *A copula based approach for design of multivariate random forests for drug sensitivity prediction*. RF, VMRF, CMRF results (5 fold cross validation) with and without prior feature selection (**Table A**). Results for CCLE Dataset drug sensitivity prediction for a drugset with 4 drugs in the form of correlation coefficients for *RF*, *VMRF*, *CMRF* and *KBMTL* approaches (**Table B**). Results for GDSC Dataset drug sensitivity prediction for a drugset with 3 drugs in the form of correlation coefficients for *RF*, *VMRF*, *CMRF* and *KBMTL* approaches (**Table C**). Results for GDSC Dataset drug sensitivity prediction for a drugset with 140 drugs in the form of correlation coefficients is shown (only 15 drugs that are common with CCLE are shown in detail while the average represents the average of all 140 drugs) (**Table D**). Results for GDSC Dataset drug sensitivity prediction for a drugset with 140 drugs in the form of NRMSE is shown (only 15 drugs that are common with CCLE are shown in detail while the average represents the average of all 140 drugs) (**Table E**). Results for CCLE Dataset drug sensitivity prediction for the combined set of 24 drugs in the form of correlation coefficients (**Table F**). Results for CCLE Dataset drug sensitivity prediction for the combined set of 24 drugs in the form of Normalized Root Mean Square Error (**Table G**). Comparison of *α* for different sets of synthetic data with and without noise added to the drug response (**Table H**). Comparison of *α* for different amount of random subset of the original samples in a specific synthetic data. Original number of samples were 350 in this specific example (**Table I**). Simulation time for different drugsets in GDSC data. The reported simulation times are the time needed to generate complete result for all drugs in a drug set for 5 fold cross validation (**Table J**). Simulation time for different drugsets in GDSC data. The reported simulation times are the time needed to generate complete result for all drugs in a drug set for 30–70 case (**Table K**). Simulation time for different methods for all drugs of GDSC dataset (140) and CCLE dataset (24). The reported simulation times are the time (in seconds) needed to generate complete result for all drugs for 30–70 case (**Table L**).
(PDF)
