# Supplementary material for: A Copula Based Approach for Design of Multivariate Random Forests for Drug Sensitivity Prediction
Source: PLoS One. 2015 Dec 10;10(12):e0144490. doi: 10.1371/journal.pone.0144490 (PMC4684346; doi:10.1371/journal.pone.0144490)
Supplement: S1 File — RF, VMRF, CMRF results (5 fold cross validation) with and without prior feature selection (Table A). Results for CCLE Dataset drug sensitivity prediction for a drugset with 4 drugs in the form of correlation coefficients for RF, VMRF, CMRF and KBMTL approaches (Table B). Results for GDSC Dataset drug sensitivity prediction for a drugset with 3 drugs in the form of correlation coefficients for RF, VMRF, CMRF and KBMTL approaches (Table C). Results for GDSC Dataset drug sensitivity prediction for a drugset with 140 drugs in the form of correlation coefficients is shown (only 15 drugs that are common with CCLE are shown in detail while the average represents the average of all 140 drugs) (Table D). Results for GDSC Dataset drug sensitivity prediction for a drugset with 140 drugs in the form of NRMSE is shown (only 15 drugs that are common with CCLE are shown in detail while the average represents the average of all 140 drugs) (Table E). Results for CCLE Dataset drug sensitivity prediction for the combined set of 24 drugs in the form of correlation coefficients (Table F). Results for CCLE Dataset drug sensitivity prediction for the combined set of 24 drugs in the form of Normalized Root Mean Square Error (Table G). Comparison of α for different sets of synthetic data with and without noise added to the drug response (Table H). Comparison of α for different amount of random subset of the original samples in a specific synthetic data. Original number of samples were 350 in this specific example (Table I). Simulation time for different drugsets in GDSC data. The reported simulation times are the time needed to generate complete result for all drugs in a drug set for 5 fold cross validation (Table J). Simulation time for different drugsets in GDSC data. The reported simulation times are the time needed to generate complete result for all drugs in a drug set for 30–70 case (Table K). Simulation time for different methods for all drugs of GDSC dataset (140) and CCLE dataset ( [file pone.0144490.s001.pdf]

S1: Supplementary Information for Article: *A copula based approach for design of multivariate random forests for drug sensitivity prediction*

Saad Haider[1], Raziur Rahman[1], Souparno Ghosh[2], Ranadip Pal[1,*],

1 Department of Electrical and Computer Engineering, Texas Tech University, Lubbock, Texas, United States
2 Department of Mathematics and Statistics, Texas Tech University, Lubbock, Texas, United States

*** Corresponding Author: ranadip.pal@ttu.edu**

Changes in performance with prior feature selection

Random forest (RF) is designed to create uncorrelated trees using random subsets of features in each node of each tree. RF by itself is a great tool for feature selection from a high dimensional set of features. But we observed that the prediction accuracy is improved when a prior feature selection (RELIEFF) [1] approach is implemented. Table A shows the performance of RF, VMRF and CMRF with and without RELIEFF feature selection in 2 drug sets of GDSC.

Table A: RF, VMRF, CMRF results (5 fold cross validation) with and without prior feature selection

			With RELIEFF			Without RELIEFF		
Drug Set	Common Target	Drug Name	*RF*	*VMRF*	*CMRF*	*RF*	*VMRF*	*CMRF*
S_{C1}	EGFR	Erlotinib	0.5156	0.5193	0.5301	0.4093	0.4312	0.4384
		Lapatinib	0.5544	0.5742	0.5699	0.4747	0.4722	0.4881
S_{C2}	ABL1	AZD-0530	0.3553	0.3810	0.3990	0.1968	0.1919	0.2124
		TAE-684	0.4060	0.4100	0.4338	0.2216	0.2692	0.2684

Performance Analysis for drugsets consisting of more than two drugs

We have generated empirical copulas for the bivariate cases as they are able to capture all forms of dependency structures. However, generation of empirical copulas has high computational complexity along with the need for a significant number of training samples at each node. Thus for more than two drug responses, we have considered parametric copulas and the difference between Gaussian copula parameters generated using root node and split node samples instead of the integral difference between empirical copulas is used. To test our hypothesis that VMRF and CMRF will perform better than RF, we considered a drug set with 4 different drugs from CCLE with single common target between them and a drug set with 3 different drugs in GDSC with a common target between them. The CCLE set has 482 cell lines and the GDSC set has 308 cell lines. RELIEFF was used to reduce the feature space prior to random forest

application. For simplicity, in this case, we've used 30% of the sample cell lines as training data and 70% of them as testing data.

The CCLE drugset is $S_M = \{$ *Erlotinib, Lapatinib, ZD6474(Vandetanib) , AZD0530(Saracatinib)* $\}$ with $EGFR$ as a common target [2–5]. The correlation coefficient of the experimental and predicted response of the testing data are shown in Table B.

Table B: Results for CCLE Dataset drug sensitivity prediction for a drugset with 4 drugs in the form of correlation coefficients for RF, $VMRF$, $CMRF$ and $KBMTL$ approaches.

			Correlation Co-efficients			
Drug Set	Common Target	Drug Name	RF	$VMRF$	$CMRF$	$KBMTL$
S_M	EGFR	Erlotinib	0.3533	0.3604	0.3791	0.2576
		Lapatinib	0.4142	0.4396	0.4001	0.2682
		ZD-6474	0.2067	0.1975	0.1907	0.1583
		AZD-0530	0.1419	0.1539	0.1818	0.1120

Table B shows that the average correlation coefficient of these 4 drugs are higher for CMRF (0.2879) and VMRF (0.2878) as compared to RF(0.2790) in spite of RF performing better for couple of these drugs. All three random forest based approaches outperforms KBMTL in terms of correlation coefficients in this scenario.

Table C shows the predictive performance for GDSC dataset for a drugset with 3 drugs (AZD-0530, Erlotinib, Lapatinib) with common target $EGFR$. The average correlation coefficient of these 3 drugs is highest in KBMTL (0.5547) followed by CMRF (0.5130), VMRF (0.5116) and RF (0.5053).

Table C: Results for GDSC Dataset drug sensitivity prediction for a drugset with 3 drugs in the form of correlation coefficients for RF, $VMRF$, $CMRF$ and $KBMTL$ approaches.

			Correlation Co-efficients			
Drug Set	Common Target	Drug Name	RF	$VMRF$	$CMRF$	$KBMTL$
S_M	EGFR	AZD-0530	0.5869	0.5875	0.5934	0.5555
		Erlotinib	0.4755	0.4801	0.5069	0.6615
		Lapatinib	0.5316	0.5493	0.5508	0.4470

We have also applied the methodology to the complete dataset consisting of 140 drugs in GDSC where joint prediction of 140 drugs is conducted for VMRF, CMRF and KBMTL. The correlation coefficients for 15 of the drugs that are common with CCLE dataset along with the average of all 140 drugs are shown in Table D while Table E shows the performance in terms of NRMSE. In terms of average correlation coefficients, RF performs the best followed by $VMRF$, $CMRF$ and $KBMTL$. In terms of NRMSE, the average performance of $VMRF$ and $CMRF$ is similar followed by RF and $KBMTL$. It appears that for large number of drugs with minimal relationships among the drugs, univariate RF is often the better choice for average performance.

Tables F and G shows the performance in the form of correlation coefficients and NRMSE for predicting jointly the 24 drugs in CCLE dataset. Similar to GDSC case scenario, RF performs the best followed by $VMRF$, $CMRF$ and $KBMTL$ in terms of correlation coefficients. In terms of NRMSE, the average performance of RF is the best followed by KBMTL, VMRF and CMRF. The CCLE dataset also lends support to the conclusion that univariate random forest can outperform the Multivariate approaches when there is limited relationship among the drugs. In terms of time taken for simulation of large set of drugs, $VMRF$ is the fastest followed by RF, $CMRF$ and $KBMTL$ as shown later in Table L.

Table D: Results for GDSC Dataset drug sensitivity prediction for a drugset with 140 drugs in the form of correlation coefficients is shown (only 15 drugs that are common with CCLE are shown in detail while the average represents the average of all 140 drugs).

			Correlation Coefficients			
Drug Set	Common Target	Drug Name	RF	$VMRF$	$CMRF$	$KBMTL$
S_M	None	17-AAG	0.6209	0.6283	0.5798	0.2446
		AZD-0530	0.1132	0.0899	0.0881	0.1297
		AZD6244	0.3709	0.3976	0.3520	0.2126
		Erlotinib	0.4732	0.3914	0.4096	0.1802
		Lapatinib	0.3041	0.3477	0.3865	0.0866
		Nilotinib	0.2926	0.2101	0.1936	0.2599
		Nutlin-3	0.0482	0.0130	-0.0428	0.2297
		Paclitaxel	0.1632	0.1280	0.1561	0.1176
		PD-0325901	0.4097	0.4029	0.3351	0.1751
		PD-0332991	0.1278	0.0626	0.0850	0.3678
		PF2341066	0.2401	0.1472	0.1438	0.1950
		PHA-665752	0.1111	0.0096	-0.0034	0.0117
		PLX4720	0.1918	0.1718	0.1543	0.0214
		Sorafenib	0.0707	0.0185	0.0402	0.0916
		TAE-684	0.1323	0.1872	0.0731	0.0661
Average Correlation Coefficient			0.2354	0.2120	0.1985	0.1553

Table E: Results for GDSC Dataset drug sensitivity prediction for a drugset with 140 drugs in the form of NRMSE is shown (only 15 drugs that are common with CCLE are shown in detail while the average represents the average of all 140 drugs).

			Correlation Co-efficients			
Drug Set	Common Target	Drug Name	RF	$VMRF$	$CMRF$	$KBMTL$
S_M	None	17-AAG	0.8481	0.9223	0.9258	0.9703
		AZD-0530	0.9940	0.9965	0.9965	0.9921
		AZD6244	0.9602	0.9751	0.9783	0.9787
		Erlotinib	0.9280	0.9665	0.9413	0.9842
		Lapatinib	0.9589	0.9716	0.9539	1.0009
		Nilotinib	0.9913	0.9966	0.9968	0.9901
		Nutlin-3	1.0018	1.0016	1.0033	0.9742
		Paclitaxel	0.9867	0.9932	0.9897	1.0510
		PD-0325901	0.9427	0.9675	0.9770	0.9845
		PD-0332991	0.9918	0.9980	0.9969	0.9373
		PF2341066	0.9778	0.9922	0.9915	0.9820
		PHA-665752	0.9938	1.0013	1.0014	1.0059
		PLX4720	0.9877	0.9933	0.9955	1.0047
		Sorafenib	0.9977	0.9999	0.9992	0.9977
		TAE-684	0.9917	0.9895	0.9974	1.0411
Average Normalized Root Mean Square Error			0.9735	0.9865	0.9865	1.0065

Table F: Results for CCLE Dataset drug sensitivity prediction for the combined set of 24 drugs in the form of correlation coefficients.

			Correlation Co-efficients			
Drug Set	Common Target	Drug Name	RF	$VMRF$	$CMRF$	$KBMTL$
S_M	None	17-AAG	0.2792	0.2028	0.2606	0.2159
		AZD-0530	0.3042	0.3001	0.2708	0.2839
		AZD6244	0.4978	0.3637	0.3799	0.5366
		Erlotinib	0.3682	0.3206	0.3160	0.3525
		Lapatinib	0.3073	0.3059	0.2671	0.2252
		Nilotinib	0.5471	0.5581	0.5024	0.4194
		Nutlin-3	0.3715	0.3756	0.3921	0.3211
		Paclitaxel	0.4342	0.3649	0.4444	0.4475
		PD-0325901	0.5671	0.4699	0.4482	0.5782
		PD-0332991	0.5136	0.4987	0.4988	0.4329
		PF2341066	0.4828	0.4863	0.4559	0.4439
		PHA-665752	0.4669	0.4573	0.4254	0.4169
		PLX4720	0.3750	0.3724	0.3331	0.1323
		Sorafenib	0.5162	0.5073	0.4714	0.4035
		TAE-684	0.3972	0.4035	0.3980	0.3904
		AEW541	0.4379	0.4236	0.3167	0.3646
		Irinotecan	0.6063	0.5921	0.5989	0.6305
		L-685458	0.6379	0.6233	0.6286	0.5754
		LBW242	0.0740	0.1125	0.0433	0.0951
		Panobinostat	0.7073	0.7335	0.6836	0.6472
		RAF265	0.2976	0.3177	0.2756	0.2113
		TKI258	0.5127	0.4988	0.4912	0.4148
		Topotecan	0.6016	0.5876	0.6003	0.6129
		ZD-6474	0.3089	0.3274	0.2619	0.2590
Average Correlation Coefficient			0.4422	0.4251	0.4068	0.3921

Table G: Results for CCLE Dataset drug sensitivity prediction for the combined set of 24 drugs in the form of Normalized Root Mean Square Error.

			NRMSE			
Drug Set	Common Target	Drug Name	RF	$VMRF$	$CMRF$	$KBMTL$
S_M	None	17-AAG	0.9747	0.9854	0.9805	0.9979
		AZD-0530	0.9669	0.9724	0.9740	0.9672
		AZD6244	0.9274	0.9611	0.9770	0.8526
		Erlotinib	0.9404	0.9604	0.9668	0.9405
		Lapatinib	0.9520	0.9638	0.9744	0.9777
		Nilotinib	0.8980	0.8992	0.9437	0.9204
		Nutlin-3	0.9431	0.9476	0.9650	0.9489
		Paclitaxel	0.9143	0.9457	0.9533	0.9186
		PD-0325901	0.9194	0.9613	0.9779	0.8330
		PD-0332991	0.8707	0.8934	0.9394	0.9026
		PF2341066	0.9027	0.9140	0.9543	0.9016
		PHA-665752	0.8958	0.9085	0.9490	0.9101
		PLX4720	0.9432	0.9517	0.9768	0.9964
		Sorafenib	0.9010	0.9182	0.9581	0.9162
		TAE-684	0.9470	0.9538	0.9797	0.9262
		AEW541	0.9440	0.9540	0.9854	0.9337
		Irinotecan	0.8373	0.8684	0.9256	0.7763
		L-685458	0.8125	0.8412	0.9184	0.8430
		LBW242	1.0011	0.9937	0.9995	1.0071
		Panobinostat	0.7651	0.7999	0.9039	0.7813
		RAF265	0.9589	0.9624	0.9810	0.9910
		TKI258	0.8842	0.9085	0.9502	0.9106
		Topotecan	0.8410	0.8753	0.9268	0.7919
		ZD-6474	0.9556	0.9601	0.9754	0.9749
Average Normalized Root Mean Square Error			0.9124	0.9292	0.9599	0.9134

Robustness analysis of α (Method-2) using synthetic example

At first, we have analyzed robustness of α generated using pareto frontier approach by adding noise to the drug response data and comparing with the α generated from the response without noise. This simulation was conducted using simulated data generated from the same framework mentioned in the synthetic example included in main manuscript. 4 different sets of synthetic data sets were created with different number of samples and corresponding α values are reported for with and without noise added to the drug response (table H). In all cases, we have used 30% of the sample cell lines as training data and 70% of them as testing data. Number of trees were 100 in all cases.

Table H: Comparison of α for different sets of synthetic data with and without noise added to the drug response.

		α	
Drug Set	Number of Samples	Without noise	With noise
S_1	200	6.23	7.63
S_2	250	4.53	5.05
S_3	300	4.66	5.15
S_4	350	4.43	4.95

Finally, we have analyzed the robustness by comparing α generated using different selections of random subsets of the original samples. Table I shows the α values generated from a drugset with N=350 samples and with random subset of the same data with $0.9N$, $0.8N$, $0.7N$ samples.

Table I: Comparison of αs for different selections of random subsets of the original samples in a specific synthetic dataset. Original number of samples were 350 in this particular example.

Drug Set	Number of Samples	α
S_N	N	4.43
	0.9N	4.88
	0.8N	5.16
	0.5N	0.63

Simulation Time Complexity

Simulation time of drugsets S_{C1}, S_{C2} and S_{C3} of GDSC are reported in Table J for RF, VMRF and CMRF methods. The reported simulation times are the time needed to generate complete result for all drugs in a drug set for 5 fold cross validation. Simulation was conducted in a Intel Core i7 computer with 16GB RAM. 4 labs had been used while running the simulation under MATLABPOOL.

Table J: Simulation time for different drugsets in GDSC data. The reported simulation times are the time needed to generate complete result for all drugs in a drug set for 5 fold cross validation.

		Simulation Time (seconds)		
Drug Set	Number of Samples	RF	VMRF	CMRF (Empirical)
S_{C1}	316	982	613	13850
S_{C2}	349	1110	690	15400
S_{C3}	645	2192	1282	28764

In addition to 5-fold cross validation simulation times, we are reporting the simulation times with 30% of the sample cell lines as training data and 70% of them as testing data in Table K . We are also including the CMRF (parametric) simulation time in table K . Table L shows the simulation times for predicting all drugs jointly for GDSC and CCLE datasets using RF, VMRF, CMRF (parameteric) and KBMTL approaches. Similar to Table K, we have used 30% of the cell lines as training data and 70% of them as testing data.

Table K: Simulation time for different drugsets in GDSC data. The reported simulation times are the time needed to generate complete result for all drugs in a drug set for 30-70 case.

		Simulation Time (seconds)			
Drug Set	Number of Samples	RF	VMRF	CMRF (Empirical)	CMRF (Parametric)
S_{C1}	316	169	90	734	412
S_{C2}	349	190	105	795	455
S_{C3}	645	350	190	1510	850

Table L: Simulation time for different methods for all drugs of GDSC dataset (140) and CCLE dataset (24). The reported simulation times are the time (in seconds) needed to generate complete result for all drugs for 30-70 case.

Method Name	GDSC dataset	CCLE dataset
Random Forest	3,930	833.47
Multivariate Random Forest	51.17	45.70
Parametric CMRF	21,700	1,230
KBMTL	192,000	46,400

References

1. Robnik-Sikonja M, Kononenko I. Theoretical and empirical analysis of ReliefF and RReliefF. Machine Learning. 2003;53:23–69.
2. Ling YH, Li T, Yuan Z, Haigentz M, Weber TK, Perez-Soler R. Erlotinib, an effective epidermal growth factor receptor tyrosine kinase inhibitor, induces p27KIP1 up-regulation and nuclear translocation in association with cell growth inhibition and G1/S phase arrest in human non-small-cell lung cancer cell lines. Molecular pharmacology. 2007;72(2):248–258.
3. Johnston SR, Leary A. Lapatinib: a novel EGFR/HER2 tyrosine kinase inhibitor for cancer. Drugs Today (Barc). 2006;42(7):441–453.
4. Morabito A, Piccirillo MC, Falasconi F, De Feo G, Del Giudice A, Bryce J, et al. Vandetanib (ZD6474), a dual inhibitor of vascular endothelial growth factor receptor (VEGFR) and epidermal growth factor receptor (EGFR) tyrosine kinases: current status and future directions. The oncologist. 2009;14(4):378–390.
5. Larsen AB, Stockhausen MT, Poulsen HS. Cell adhesion and EGFR activation regulate EphA2 expression in cancer. Cellular signalling. 2010;22(4):636–644.
